# Synthesis of low pH stable, highly photocatalytic alginate hydrogels for methylene blue removal from wastewater

**DOI:** 10.55730/1300-0527.3719

**Published:** 2024-12-08

**Authors:** Emircan UYSAL, Halide Nur DURSUN, Emre Can UYSAL, Sebahattin GÜRMEN

**Affiliations:** 1 Department of Metallurgical and Materials Engineering, Faculty of Chemical and Metallurgical Engineering, Istanbul Technical University, İstanbul, Turkiye; 2 Department of Mineral Processing Engineering, Faculty of Mines, Istanbul Technical University, İstanbul, Turkiye; 3 Department of Chemistry, Faculty of Science and Letters, Istanbul Technical University, İstanbul, Turkiye

**Keywords:** Hydrogel, adsorption, wastewater treatment, photocatalytic degradation

## Abstract

Chemical compounds in wastewater, including dye molecules, have been identified as significant concerns for environmental integrity and human health. In this study, adsorbent materials were synthesized from biodegradable polymers to remove methylene blue (MB) from wastewater. Calcium alginate hydrogels were produced and coated with chitosan to enhance their pH stability. Furthermore, the calcium alginate gels were doped with silver nanoparticles to improve their photocatalytic properties, promoting MB degradation and enabling gel reuse. The adsorption behavior of the gels was comprehensively examined under dark conditions, considering factors such as time, solid-to-liquid ratio, temperature, and pollutant concentration, all of which significantly impact the adsorption process. The wastewater, containing 100 ppm of MB, was subjected to a cleaning process that resulted in a reduction of the initial concentration by over 95%. Adsorption kinetics, thermodynamic constants, and adsorption isotherms were studied to understand the underlying mechanisms better. It was observed that the adsorption kinetics were compatible with pseudo-second-order with R^2^ values above 0.99 and the adsorption kinetics were compatible with Freundlich with 0.9996. Modeling and simulation studies were used to establish the correlation between adsorption behavior and the concentration of dye material. Subsequently, UV light exposure was applied, enhancing further efficiency and enabling gel regeneration. Even after five cycles, the gels maintained an efficiency of 88.42%. Structural characterizations, pH stability assessments, and cytotoxicity analyses were conducted to evaluate the suitability of the gels for wastewater treatment with minimal environmental or health impact.

## 1. Introduction

Wastewater is defined as liquid waste resulting from a variety of processes, including those associated with domestic, industrial, commercial, and agricultural activities [1]. It typically contains water and contaminants such as food particles, oils, chemicals, microorganisms, organic and inorganic materials, and heavy metals [2]. Wastewater is also generated using dyes in industrial and commercial activities, such as in textile, food, chemical, pharmaceutical, and oil companies [3]. This type of wastewater contains various organic dyes such as rhodamine, methyl orange, malachite green, and methylene blue (MB) [4]. The dye molecules are composed of a chromogen or aromatic structure that can absorb visible light, making them visible to the naked eye [5]. Nitrogen dyes are the most well-known among the 12 classes of chromogenic groups. Examples of these dyes include Orange G, Acid orange 7, New coxin, Acid black 1, Tartrazine, Acid yellow 17, Congo red, and MB, all of which contain at least one azo group (−N=N−) [3]. These dyes are known to be highly resistant to degradation from exposure to light, water, and chemicals due to their complex chemical structure. As a result, they are considered the most challenging dyes to remove from wastewater [6]. The presence of synthetic dyes in wastewater poses serious risks to the environment and human health; these dyes can cause coloration of water bodies, reduce light transmittance, have toxic effects on fish and other aquatic organisms, negatively affect soil health by disrupting microbial communities, and endanger nutrient cycling and soil fertility [7–9]. In addition, it has been reported that exposure to these dyes can lead to various health problems ranging from allergies, dermatitis, respiratory problems and cancer due to carcinogenic effects, and the mixing of wastewater containing untreated dyes into drinking water sources reduces water quality, making it unsafe for human consumption [7,8]. Due to the impact of wastewater on human health and the environment, it must be purified from these kinds of dyes.

Numerous techniques have been developed to eliminate dyes from wastewater, including precipitation, membrane processes, electrochemical processes, ion exchange, photocatalytic degradation, and adsorption [10]. The adsorption process attracts attention due to its low cost, easy operation, applicability to various dye molecules, and high efficiency [11]. Moreover, using biopolymers as adsorbent material improves the environmental impact of the process. The utilization of biopolymers in wastewater treatment is advantageous due to their distinctive characteristics, including biodegradability and biocompatibility [12]. This approach has the potential to mitigate environmental damage. Biopolymers such as alginate and chitosan have been extensively studied for their potential in adsorbing dyes from wastewater [13,14]. Alginate, extracted from seaweed, is used in wastewater treatment as beads, hydrogels, and composites due to its biodegradability, renewability, and low cost compared to synthetic dye-removal adsorbents [15]. Alginate is a biopolymer derived from seaweed and comprises two monomers: β-d-mannuronate (M) and α-l-guluronate (G) in a linear copolymer structure [16]. The chemical structure of alginate is a linear copolymer of 1,4-linked residues of β-d-M and α-l-G [17]. Alginate gelation is achieved through ion exchange between the monovalent sodium ions (Na^+^) in the alginate solution and polyvalent cations, such as calcium ions (Ca^2+^) [18]. Furthermore, the obtained structure, due to these ionic interactions, is called hydrogel, and in studies conducted with alginate hydrogels, very high efficiencies of dye removal from wastewater have been achieved. Asadi et al. [19] tested the removal of methyl violet in wastewater with the calcium alginate hydrogels they produced and calculated the adsorption capacity of the gels as 889 mg g^−1^ under optimum conditions.

Moreover, calcium alginate gels exhibit low stability at low pH due to the protonation of carboxyl groups that interact with Ca^2+^, which causes structural degradation. In cases such as low pH that may occur during the storage of the hydrogels, these materials must maintain their stability. The pH stability of alginate gels can be increased by coating gels with chitosan. A common and plentiful polymer found in nature, chitosan, which is the completely or partially deacetylated form of chitin, provides the fundamental supporting framework for a wide variety of living things, including insects, arthropods, fungi, and crustaceans [20]. Chitosan is a cationic polymer with primary amine groups in its polymeric backbone [21]. Deacetylation of chitin yields chitosan, which has both deacetylated and undeacetylated groups [12]. Moreover, chitosan is a polymer of N-acetyl glucosamine that has been partially deacetylated [22]. When chitosan and alginate are combined, these polymers form polyelectrolyte complexes because of their oppositely charged polymeric backbones. These complexes provide better mechanical and chemical stability than the individual compounds [23].

In addition to the adsorption process, the photocatalytic degradation of dyes in wastewater provides more efficient treatment. This process consists of two steps: the adsorption of dye molecules onto the adsorbent surface and the subsequent decomposition of the adsorbed molecules by light irradiation. Photocatalytic degradation not only increases the adsorption efficiency but also enables the reuse of the adsorbent material after its surface has been cleaned by photocatalytic degradation; reusing adsorbent materials is very important from an environmental and economic perspective. Furthermore, the fact that the adsorbent material has photocatalytic properties is essential in this process. As individual biopolymers, alginate [24] and chitosan [25] do not have inherent photocatalytic properties. In addition to adsorption studies with this polymer, different materials can be doped to provide photocatalytic properties. Copper(II) oxide (CuO) [26], zinc oxide (ZnO) [27], titanium dioxide (TiO_2_) [28], tin oxide [29], cerium oxide [30], and silver (Ag) [31] nanoparticles (NPs) are examples of materials that have photocatalytic properties. Even though Ag NPs are in metallic form, because of the surface plasmon resonance (SPR) effect, they can have a photocatalytic impact [32]. Moreover, since these metallic NPs are unstable due to agglomeration [31], they need to be functionalized, and by doping them into a gel matrix, highly efficient photocatalytic degradation can be achieved. Hasan et al. [31] prepared poly(acrylonitrile), alginate, and Ag nanocomposites and reported the removal of 2,4-dinitrophenol in wastewater by adsorption and photocatalytic degradation. They reported reaching 99.46% photocatalytic capacity with the nanocomposites they produced. The results obtained indicate that alginate and Ag NPs are effective in the removal of dyes from wastewater.

The current study focused on functionalized calcium alginate gels developed for the removal of MB from wastewater. Unlike conventional alginate-based adsorbents, the gels in this study were coated with chitosan to provide long-term stability under low pH conditions, which is a critical advantage for industrial applications and storage. In addition, the incorporation of Ag NPs synthesized via chemical reduction into the gels provided the gels with dual functionality for both adsorption in the dark and photocatalytic degradation under ultraviolet visible (UV-vis) light. A macro-sized gel format was designed to increase scalability, and structural characterization confirmed the successful integration of these functional components. In particular, the gels maintained their stability for 30 days in low pH environments (around 90%), which is a significant improvement over similar materials reported in the literature. The kinetic and isothermal models of the dark adsorption reaction were optimized, and cycling analyses showed that the gels maintained their performance in reuse. Furthermore, cytotoxicity analyses confirmed that the gels are biocompatible and suitable for environmental applications. These innovations highlight the novel aspects of the work, particularly in addressing challenges such as stability, multifunctionality and scalability.

## 2. Materials and methods

### 2.1. Materials

Diethanol amine (as a reducing agent and solvent; Merck KGaA, Darmstadt, Germany), polyacyrlic acid (as a surface active material, M_w_ = 1800 mol/g; Sigma-Aldrich Chemical Co., St. Louis, MO, USA), and Ag nitrate (as precursor material; Merck KGaA) were used for the production of the Ag NPs. Furthermore, sodium alginate (ISOLAB Laborgeräte GmbH, Eschau, Germany) was used as the starting material, Ca chloride (CaCl_2_) (E–401; Kimbiotek Kimyevi Maddeler San. Tic. A.Ş., Türkiye) was used as a cross-linking agent for hydrogel production, and chitosan (Sigma-Aldrich Chemie GmbH, Buchs, Switzerland) was used to increase the pH resistance of the gels. Various quantities of MB (Merck KGaA) were used to prepare the synthetic wastewater. Moreover, 2% acetic acid (Merck KGaA), acetone (99.5%; Tekkim Kimya Ltd. Sti.), ethanol (Merck KGaA), nitric acid (HNO_3_) (65%; Tekkim Kimya Ltd. Sti., Bursa, Türkiye), sodium hydroxide (NaOH) (Tekkim Kimya Ltd. Sti.), and deionized water (produced from Milli-q) were used at different stages.

### 2.2. Synthesis hydrogels

The method for producing the Ag NPs was replicated in our previous study [32]. Similar to that study, the hydrogel was prepared, and the gels were doped with NPs and coated with chitosan [12]. Briefly, 0.4 g of sodium alginate powder was mixed with 20 mL of deionized water at 60 °C and 120 rpm using a heating-magnetic stirrer (model: MS300HS-MTOPS) until homogeneity. It was then left to allow the gases trapped in the sodium alginate solution to release for 24 h at room temperature. Various amounts of Ag NPs were added by weight to 20 mL solutions and homogenized using a Hielscher UP200HT ultrasonic homogenizer (Hielscher Ultrasonics GmbH, Teltow, Germany). The amount of Ag NPs contained in the gels and gel codes are given in Table 1. Droplets of 100 μL of the homogenized solution were added to 50 mL of 0.1 M CaCl_2_ solution to allow gelation of the alginate with Ca^2+^, using a magnetic stirrer. The gels were stirred for 2 h. Then, they were filtered and washed five times with deionized water. The hydrogels were kept at room temperature for 24 h to complete gelation. The stability of their pH was achieved by coating them with chitosan, and they were then stirred for 2 h in 50 mL of 2% acetic acid solution containing 1 g of chitosan. They were subsequently washed seven times with deionized water and allowed to remain at room temperature for 24 h. The experimental scheme is given Figure S1.

### 2.3. Adsorption experiments

First, 20 mL solutions containing 100, 10, and 5 ppm of MB were prepared, and the adsorbent material was stirred with a magnetic stirrer at 500 rpm and pH 7. The effects of the essential parameters affecting the adsorption reaction, which are time, chitosan coating, temperature, initial MB concentration, and the amount of adsorbent, as outlined in Table S1, were investigated. The optimal adsorption conditions were identified, and adsorption experiments were conducted under UV light. All the adsorption experiments conducted before UV light exposure were performed in a dark environment, shielded from sunlight. Adsorption efficiencies of the experiments were calculated using Eq. (1):


(1) $$\text{Adsorpsiton effiency }(\%)=\left(\frac{C_{0}-C_{e}}{C_{0}}\right)\times 100$$

Here, C_0_ is the initial concentration of the MB solution (g L^−1^), and C_e_ is the concentration at equilibrium (g L^−1^). The concentration was calculated using UV-vis analysis, and the calibration line was used in concentration calculations. It was observed that the MB peaked at 660 nm, and the concentration was calculated from the absorbance values of the peak of the solutions at 660 nm. Furthermore, the adsorbent capacity of the gels (qt) was calculated using Eq. (2):


(2) $$q_{t}=(C_{0}-C_{t})\times \frac{V}{m}$$

Here, q_t_ is the adsorbent capacity at a certain time (mg g^−1^), C_t_ is the solution concentration at a certain time (mg mL^−1^), V is the solution volume (mL), and m is the amount of adsorbent (g).

#### 2.3.1. Adsorption kinetics

The first adsorption experiment was carried out with 1 g of gel and 20 mL of 100 ppm MB solution at 20 °C and pH 7 for 1 h with C1AgCAlg, C5AgCAlg, C10AgCAlg, and 10AgCaAlg. Samples were taken from the solution at certain intervals during these adsorption reactions to determine adsorption kinetics. Determining adsorption kinetics is essential to predict the results of the adsorption reaction to be conducted under different experimental conditions [33]. The kinetics of the adsorption reaction were determined by fitting two other models with experimental data. With Eq. (3), the compatibility of the experimental data with the linearized version of the pseudo-first-order was examined [34]:


(3) $$Ln(q_{e}-q_{t})=Lnq_{e}-K_{1}t$$

Here, q_e_ is the adsorbent calculated at equilibrium (mg g^−1^), K_1_ is the kinetic model constant, and t is the time (min). Adsorption reactions compatible with the pseudo-first-order occur due to physical and weak interactions. Moreover, the compatibility of the experimental data with the linearized version of the pseudo-second-order was examined using Eq. (4) [34]:


(4) $$\frac{t}{q_{t}}=\frac{1}{kq_{e}^{2}}+\frac{t}{q_{e}}$$

The constants here are the same as the pseudo-first-order, and reactions compatible with pseudo-second-order are considered chemically controlled (chemisorption).

#### 2.3.2. Modelling of adsorption reaction

Modeling and simulation studies were conducted to enhance the comprehension of the correlation between solution concentration and adsorption efficiency. COMSOL Multiphysics 6.2 (COMSOL, Inc., Stockholm, Sweden), using the reaction engineering module, was used for modeling and simulation of the adsorption reactions. A continuous stirred tank reactor design was developed based on the assumption that products enter and exit the system. The reactor was assumed to be perfectly mixed. Table S2 provides the model constants. The rate of adsorption over time and the variation in surface reaction rate depending on the initial MB concentration were calculated using Eq. (5)–(8):


(5) $$v_{r}\frac{d_{c_{i}}}{dt}=\Sigma_{m}v_{f,m}c_{f,mi}-vc_{i}+V_{r}R_{i}+A_{r}R_{ads,i}$$

(6) $$v=\Sigma_{m}v_{f,m}+v_{p}$$

(7) $$\begin{array}{l} v_{p}=V_{r}\Sigma_{i}\frac{R_{i}M_{i}}{\rho_{i}}\\ R_{i}=\Sigma_{j}v_{ij}r_{j} \end{array}$$

(8) $$v_{p}=\frac{R_{g}T}{p}V_{r}\Sigma_{j}R_{i}$$

Here, *c_f,mi_* is the species molar concentration of feed inlet stream (mol m^−3^), *v_f, mi_* is the inlet rate (m^3^ s^−1^), *V_r_* is the reactor volume (m^3^), *v_p_* is the volumetric production rate (m^3^ s^−1^), *v_ij_* is the stoichiometric coefficient of species *i* in reaction *j*, *M_i_* denotes the species’ molecular weight, *p_i_* species density (kg m^−3^), and *R_i_* reaction rate of species *i* (mol m^−3^ s ^−1^).

#### 2.3.3. Adsorption thermodynamics

The Gibbs free energy (ΔG^0^) of the adsorption reaction was calculated with Eq. (9)–(10), and the enthalpy (ΔH^0^) and entropy (ΔS^0^) of the reaction were calculated with Eq. (11) [35]:


(9) $$\Delta G^{0}=RTln(55,5\times K_{d})$$

(10) $$K_{d}=q_{e}/C_{e}$$

(11) $$\text{ln}(K_{d})=\frac{\Delta S^{o}}{R}-\frac{\Delta H^{o}}{RT}$$

Here, K_d_ is the equilibrium constant of the adsorption reaction.

#### 2.3.4. Adsorption isotherm

It was necessary to determine the adsorption isotherms to investigate the interaction between the adsorbate and adsorbent. The agreement between the experimental data and the Langmuir model was examined with Eq. (12) [34]:


(12) $$\frac{1}{q_{e}}=\frac{1}{K_{L}q_{m}C_{e}}+\frac{1}{q_{m}}$$

Here, Q_m_ is the maximum adsorbent capacity (g mg^−1^), C_e_ is the equilibrium solution concentration (g L^−1^), and K_L_ is the model constant. Adsorption reactions compatible with the Langmuir model occur homogeneously and in monolayers. Furthermore, the compatibility of the experimental data with the Freundlich Isotherm model was examined with Eq. (13) [34].


(13) $$\text{log}(q_{e})=\frac{1}{n_{f}}\text{log}(C_{e})+\text{log }(K_{f})$$

Here, K_f_ is the Freundlich constant, and n_f_ is the Freundlich isotherm constant.

### 2.4. Photocatalytic degradation of the MB

Photocatalytic degradation of the MB was performed in a dark box without sunlight, with a UV-vis lamp (Vnasky 100 W UV black light UV projector with a wavelength of 395 nm) and a magnetic stirrer. After adsorption was completed in the dark, photocatalytic degradation of the MB was performed. The experiments performed are summarized in Table S3. The effect of these parameters was examined. Experiments were carried out for 1 h, with 20 mL of solution and pH 7.

### 2.5. Characterization

To better examine the chemical structure of the gels, they were dried in a vacuum atmosphere at 80 °C and then Fourier-transform infrared spectroscopy (FTIR) (400–4000 cm^−1^; Bruker Corp., Billerica, MA, USA), X-ray diffraction (XRD) (Miniflex; Rigaku Corp., Tokyo, Japan; Cu *Kα*, 10° ≤ 2θ ≤ 90°), and scanning electron microscopy (SEM)/energy dispersive spectroscopy (EDS) (FEI Quattro analytical scanning electron microscope; Thermo Fisher Scientific Inc., Waltham, MA, USA) analyses were performed. To calculate the quantity of water retained by the gels throughout the production stage, they were dried at 80 °C for 6 h, and the weight change was calculated using Eq. (14):


(14) $$\text{Water Content }(\%)=\left(\frac{W_{0}-W_{t}}{W_{0}}\right)\times 100$$

Here, W_0_ (g) is the initial weight of the gels, and W_t_ (g) is the weight after the drying process. Porosity and density of the gels were calculated using Archimedes’ Principle. Point of zero charge (pH_PZC)_ values of the 1AgCaAlg, 10AgCaAlg, and C10agCaAlg gels were calculated to examine zeta potential behavior changes with increasing the amount of Ag NPs and chitosan coating. First, 40 mL of 0.1 M NaNO_3_ solution was prepared, and the pH was adjusted to 2, 4, 6, 8, 10, and 12 with 0.1 M NaOH and HNO_3_ solutions. Then, 4 g of gel was added to the solutions and shaken for 24 h at 24 °C. Next, the pH values of the solutions were measured. The initial and final pH diagram was drawn, and it was determined that the part below the intersection point of the line, where x = y, had a positive zeta potential value, while the part above had a negative zeta potential value. The band gaps of the chitosan-coated gels were calculated using Tauc’s equation, as shown in Eq. (15)[36]:


(15) $${\left(\alpha hv\right)}^{n}=B(hv-E_{g})$$

The index n (equals 2) indicates the kind of electronic transitions generating the absorption, whereas B and hv represent the constants related to the incident photon energy and the extent of band tailing, respectively [36].

#### 2.5.1. Cycle analysis of the hydrogels

Under optimized conditions, 2 g of 10AgCAlg gel was added to 10 mL of solution containing 10 ppm of MB, and mixed at pH 7 at 20 °C, in the dark, and under UV light for 1 h, and the process was carried out for five cycles.

#### 2.5.2. Long-term stability analysis of the hydrogels

To examine the stability of the 1AgCaAlg, 5AgCaAlg, 10AgCaAlg, C1AgCaAlg, C5AgCaAlg, and C10AgCaAlg gels at low pH, 1 g of each gel was kept separately in 10 mL of 0.1 M HNO_3_ solution for 30 days. Since alginate releases water in its structure and shrinks at low pH, the first weight change was taken on day 2, when it completely lost its water, and the last measurement was taken on day 30. It was observed that the physical stability of the low pH structure was lost, and it was predicted that weight measurement at the macro level could give an idea about the stability. The stability of the structure was calculated with Eq. (16):


(16) $$\text{Stability }(\%)=\left(\frac{M_{0}-M_{t}}{M_{0}}\right)\times 100$$

Here, M_0_ (g) is the initial weight of the gels, and M_t_ (g) is the weight at a certain time.

#### 2.5.3. Cytotoxicity analysis of the hydrogels

Similar to the literature, *Escherichia coli* bacterial cultures were cultured in a Petri dish using diffusion techniques for toxicity studies [37]. The original MB solutions (60 and 120 ppm) and purified hydrogel samples were freeze-dried, and the resulting residues were diluted with 0.5 and 1 mL of water, respectively. This dilution process aimed to achieve final solutions with concentrations equivalent to 60 and 120 ppm, respectively. Each sample was subjected to analysis three times. Statistical analysis was performed using the Statistical Package for the Social Sciences (SPSS), utilizing one-way analysis of variance (ANOVA) followed by post hoc pairwise analysis. Significant differences between values were evaluated using Tukey’s test at p = 0.05.

## 3. Result and discussion

### 3.1. Structural characterization of the gels

**Figure 1:**
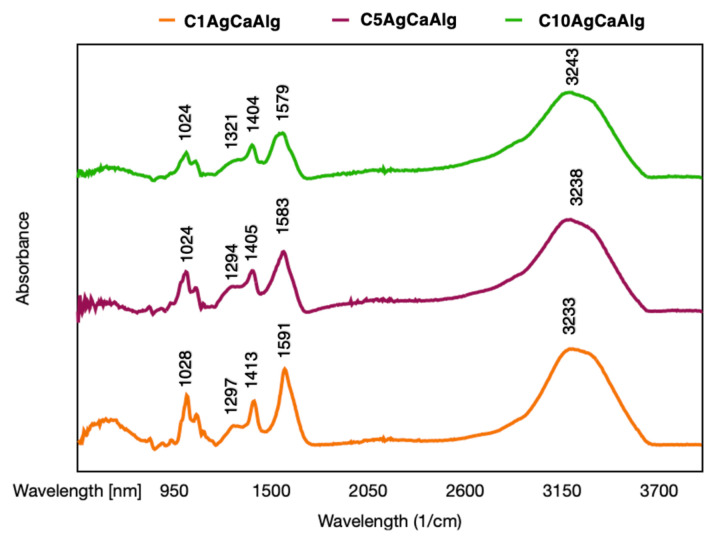
FTIR pattern of the chitosan-coated gels.

**Figure 2:**
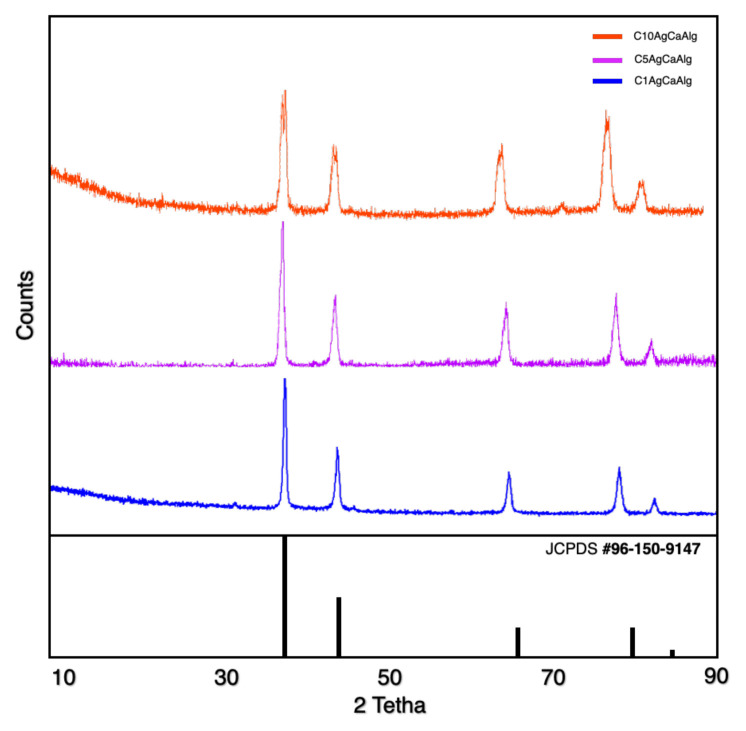
XRD pattern of the chitosan-coated gels.

FTIR analysis was conducted to determine the chemical structure of the gels produced, and the patterns are presented in Figure 1. The precursor materials, sodium alginate, and chitosan, were characterized in our previous work [12]. Three distinct gels coated with chitosan appeared to have a peak approximately at 3200 cm^−1^; this occurs due to the stretching of O-H and N-H bonds [38]. The presence of peaks around 1300 cm^−1^ (C–N stretching of amide III) and 1580 cm^−1^ (N–H bending of primary amine) was observed [39]. The peaks around 1400 cm^−1^ are related to the bending of the H-C-H [12]. C-O-C bonds peak at approximately 1100 cm^−1^, whereas the stretching peaks of C-O bonds are observed at around 1000 cm^−1^ [12,38]. The FTIR patterns proved that the calcium alginate structure was successfully produced and coated with chitosan, with the shift of the peaks of the amine group in the precursor material and the disappearance of the N-H group bonds in the chitosan pattern [12]. The XRD patterns of chitosan-coated gels are presented in Figure 2. All the gels exhibited characteristic peaks of Ag, with no additional peaks observed.

Optic images of the gels produced without and with chitosan coating are presented in Figure S2. While the color of dried Ag NPs is dark blue, Ag NPs in sodium alginate solution appear red, and the Ag NPs appear green when calcium alginate gel production is complete. Due to SPR, Ag NPs can be physically observed through color alteration [40]. We reported that the produced particles showed different UV absorbance behavior in different liquid environments and that this occurred due to agglomeration in different liquids [32]. Although there was a degree of agglomeration, the particle size was reported to be less than 10 nm in the gels produced [32]. Furthermore, the chitosan coating resulted in the darkening of the gel color and the formation of craters on the surface. Chitosan solution has a low pH and causes some carboxyl groups in the alginate structure to be protonated [6]. This process promotes the release of water in the alginate structure and the shrinkage of the gels. During the shrinkage and water release stages, water is released from specific areas, forming craters. These craters are assumed to increase the surface area and adsorption efficiency of the gels.

**Figure 3:**
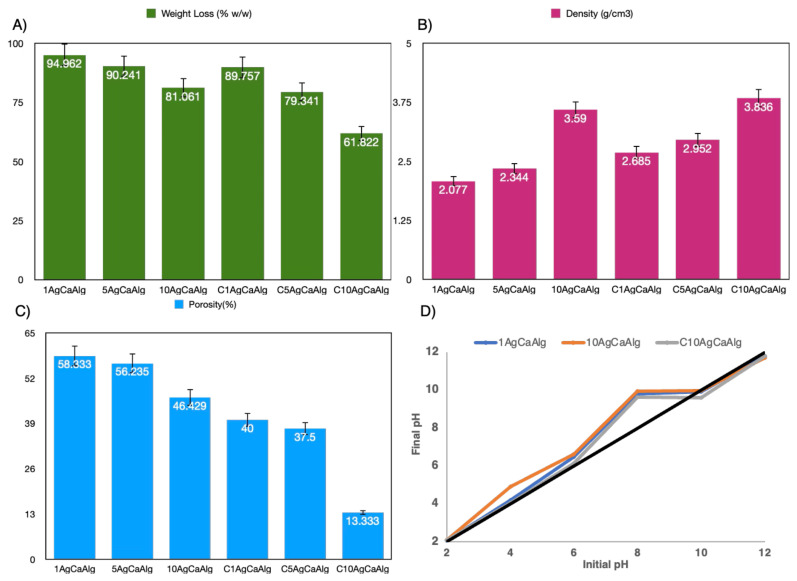
A) Percent weight loss of the gels after drying, B) density of the dried gels, C) percent of porosity of dried gels, and D) pH_PZC_ values of 1AgCaAlg, 10AgCaAlg, and C10AgCaAlg.

To determine the amount of water retained by the gels during the production phase, the gels were dried at 80 °C for 6 h, and the weight change was calculated (see Figure 3A). With increasing amounts of Ag NPs, the amount of water trapped in the gels during the production phase decreased. This phenomenon is attributed to the hydrophobic nature of Ag NPs [41]. In our prior research, different amounts of Ag Nps were added to alginate gels cross-linked with different metal ions and the amount of water trapped in the structure increased with increasing Ag concentration [32]. The prior research used the pouring technique to conduct cross-linking within a static environment. Although doping Ag NPs to alginate structure can trap more water during production by increasing the distance between the alginate chains, in the current study, the beads were produced using a magnetic stirrer and the effect of increasing the distance between the chains in the structure disappeared. At the same time, the hydrophobic property of Ag NPs became dominant. In addition, coating the gels with chitosan reduced the amount of water trapped during production, which was attributed to some protonation of the −COO^−^ groups of the alginate and shrinkage of the structure due to the low pH of the chitosan solution [12,42]. The density of the dried gels was calculated and presented in Figure 3B. The density increased as predicted with an increasing amount of Ag NPs due to the high density of the Ag. Similarly, the coating of alginate beads with chitosan makes the structure denser because the polyanion groups in the structure of alginate and the polycation groups in the structure of chitosan form a complex [43]. The amount of porosity per weight of the gels is given in Figure 3C. The porosity of the dried gels decreased with the increasing amount of Ag NPs and chitosan coating. Because of the increasing amount of Ag NPs and chitosan coating, the structure became denser, and the amount of water trapped by gels decreased. Furthermore, the pH_PZC_ values of the 1AgCaAlg, 10AgCaAlg, and C10agCaAlg gels were calculated and given in Figure 3D. Although the Ag NPs exhibited a negative surface potential after pH 3 [44], and the surface potential of the alginate molecule was negative between pH 2 and 12 [45], it was observed that all the gels produced had a positive value above around pH 7 and a negative value below pH 10. While the Ca^2+^ were influential on the surface of the gels below pH 7, they became positively charged at a pH above 10 due to a reaction between the −OH groups on the surface and the OH ions in the solution, leading to the formation of negatively charged O^−^ functional groups [46]. The gel exhibited a buffering effect between pH 7 and 10; the H^+^ and OH^−^ ions added to the solution did not affect the equilibrium pH [47]. The gels reached the point of zero charge in this region [47]. Furthermore, increasing the amount of Ag NPs in the gels and coating them with chitosan did not cause a significant change in the pH_PZC_ values. UV spectra of the chitosan-coated gels are presented in Figure S3a, and the photon energies of the C1AgCaAlg gel, calculated using the Tauc’s equation, are illustrated in Figure S3b. A peak at approximately 410 nm was identified in the spectra of the gels, which was attributed to the SPR of the Ag NPs [32]. The band gaps of the C1AgCaAlg, C5AgCaAlg, and C10AgCaAlg gels were calculated as 3.61, 3.55, and 3.48 eV, respectively.

**Figure 4:**
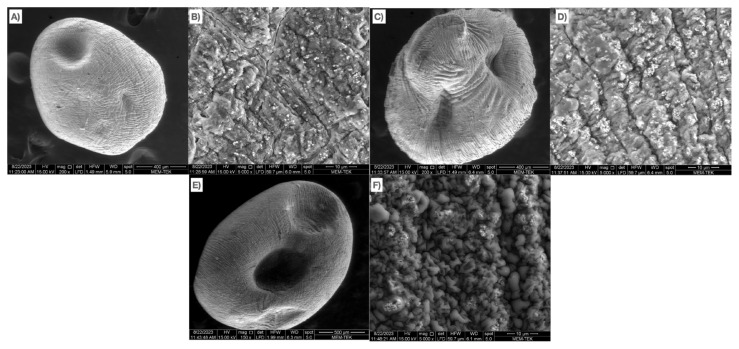
SEM images of the gels: a) C1AgCaAlg at 500X magnification, b) C1AgCaAlg at 2000X magnification, c) C5AgCaAlg at 500X magnification, d) C5AgCaAlg at 2000X magnification, e) C10AgCaAlg at 500X magnification and f) C10AgCaAlg at X2000 magnification.

**Figure 5:**
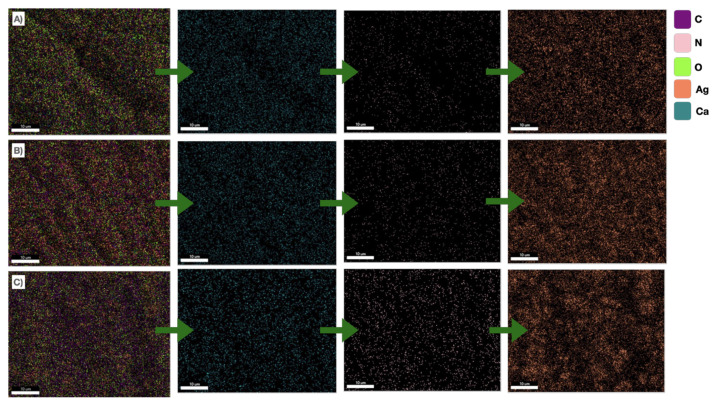
EDS maps of the gels: a) C1AgCaAlg, b) C5AgCaAlg, and c) C10AgCaAlg.

SEM images of the chitosan-coated gels with three different Ag concentrations are presented in Figure 4. Although it is possible to state that all three gels exhibited high surface roughness, increasing the concentration of Ag NPs enhances the surface roughness. The surface roughness of the gel containing 10% Ag NPs was quite apparent compared to the other gels (see Figures 4B, 4D, and 4F). Similarly, Dai et al. [48] stated that incorporating TiO_2_ NPs into alginate aerogels increased surface roughness. The increasing concentration of Ag NPs caused surface roughness due to the NPs being trapped within the alginate chemical chains. The concentration of NPs between the chains increased as the Ag NPs increased. Due to the increased trapped Ag NPs, cross-linking homogeneity decreased, and the surface roughness increased. SEM images were processed using computer-aided software, and the surface morphology of the gels was obtained (see Figure S4). As the concentration of Ag increased, the surface indentation length was accompanied by a decrease in surface homogenization. Furthermore, the EDS maps of the gels are given in Figure 5. The EDS analysis showed that as the Ag NPs concentration increased, the Ag NPs partially agglomerated on the surface. This effect resulted in the reduction of the surface protrusions while decreasing the homogenization of the surface. Although some agglomeration was observed in the gels containing 10% Ag NPs, the Ag distribution was homogeneous in all three gels. The homogeneous distribution of Ca^2+^ indicates that the alginate was successfully cross-linked, and the presence of N indicates that the cross-linked alginate was successfully coated with chitosan. Moreover, the absence of Na atoms in the EDS results shows that the NaCl byproduct formed in the washing step was successfully removed from the structure.

### 3.2. Adsorption results

#### 3.2.1. Adsorption time

**Figure 6:**
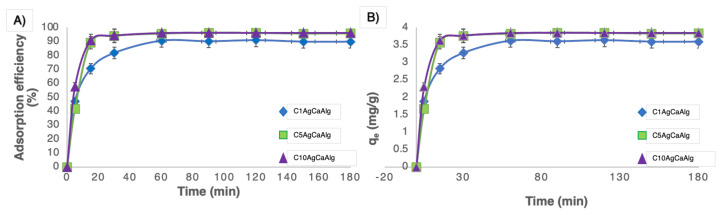
A) Adsorption efficiencies and B) adsorbent capacities of the gels (1 g of gel with varying concentrations of Ag NPs and 20 mL of pH 7 solution containing 100 ppm of MB).

The determination of the time at which the system reaches equilibrium in adsorption processes is essential for kinetic modeling and process optimization. To determine the time required to reach equilibrium for the adsorption process, 1 g of gels with varying concentrations of Ag NPs and 20 mL of pH 7 solution containing 100 ppm of MB were stirred using a magnetic stirrer at 200 rpm. The time-dependent calculated adsorption efficiency is presented in Figure 6A. While it was observed that the C1AgCaAlg gel reached equilibrium in the 60th min, the C5AgCaAlg and C10AgCaAlg gels reached equilibrium in approximately the 30th min. Increasing the concentration of Ag NPs resulted in the gels having a greater surface roughness, which shortened the time to reach equilibrium in the adsorption process. Furthermore, the adsorption efficiency for the C1AgCaAlg, C5AgCaAlg, and C10AgCaAlg gels was calculated as 90.59, 95.67, and 96.17, respectively. Increasing the roughness of the surface resulted in a reduction in equilibrium time and an enhancement in adsorption efficiency. Roughening the surface of a material leads to an extension of the surface area of the material. Expanding the surface area improves the adsorption capacity of the material in processes conducted under the same conditions as pressure and temperature [49]. Additionally, the q values of the gels were calculated against time and are presented in Figure 6B. The q_e_ values were calculated as 3.62, 3.82, and 3.84 mg g^−1^ for the C1AgCaAlg, C5AgCaAlg, and C10AgCaAlg gels, respectively. The crucial fact is that the maximum adsorption capacity that can be achieved under these conditions was calculated to be 4 mg g^−1^. In previous studies with alginate-based gels, attempts were made to exceed this value [50–52]. However, in the current study, the adsorbent capacity was limited as the objective was to degrade at high concentrations of MB molecules under UV light.

#### 3.2.2. Adsorption kinetics

**Figure 7:**
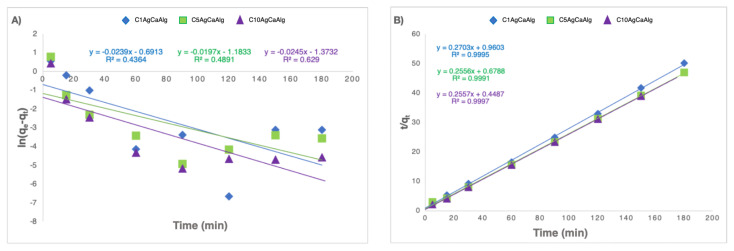
Adsorption reactions were compatible with three different gels with pseudo-first-order (A) and second-order (B).

To obtain a better understanding of adsorption processes, it is essential to understand the thermodynamics and kinetics of the reactions involved, and many vital parameters, including the time taken for the reaction to complete, can be derived from the analysis of the kinetics [53]. Figure 7 represents the agreement of experimental study results with two different kinetic models. In addition, R^2^, q_e_, and reaction constants of the pseudo-first and second-order models calculated for the three different gels are given in Table 2. It was understood that the adsorption process in the three gels was compatible with pseudo-second-order, and the adsorption process was chemisorption controlled. In the study conducted by Mundkur et al. [54], they reported that the adsorption reaction kinetics were compatible with pseudo-second-order for the MB adsorption study with clay alginate gels.

#### 3.2.3. Effect of chitosan coating on adsorption reaction

Both chitosan [55] and MB [56] are also positively charged compounds when dissolved in water. However, alginate is a negatively charged polymer. As negatively charged alginate is coated with a positively charged polymer, the ionic interaction between the alginate and MB may decrease. To observe the alteration in adsorption efficiency, adsorption studies were conducted on the 10AgCaAlg gels that were not coated with chitosan. The adsorption experiment was carried out with 1 g of gel and 20 mL of 100 ppm MB solution at 20 °C, pH 7 for 2 h. The adsorption efficiency was calculated as 96.74% (see Figure S5a), and the qe of the gels was calculated as 3.88 mg g^−1^ (see Figure S5b). The chitosan coating did not negatively affect the adsorption efficiency of the gels or the q_e_ values. Furthermore, the kinetics of the adsorption reaction was examined and is presented in Figures S5c and S5d. It was observed that gels not coated with chitosan also conformed to the pseudo-second-order, chemisorption was adequate, and the adsorption kinetics did not change with coating with chitosan.

#### 3.2.4. Effect of temperature and the thermodynamical constant of the reaction

To investigate the effect of temperature on the adsorption reaction, 20 mL solution with 100 ppm of MB and 1 g of C5AgCaAlg gel were stirred for 1 h at the temperatures in Table 3. It was observed that the adsorption efficiency significantly decreased as temperature increased. It has been reported that under typical circumstances, an increase in temperature leads to greater mobility of MB ions, increased interaction with adsorption sites, and improved adsorption efficiency [57–59]. However, the reaction herein was exothermic; therefore, the efficiency decreased with increasing temperature. In addition, another adsorption reaction was carried out under the same conditions at 5 °C to observe the adsorption efficiency at lower temperatures. The adsorption reaction efficiency was calculated as 50.94%. Although lowering the temperature should thermodynamically increase the efficiency, the reaction efficiency decreased due to the reduced mobility of the dissolved MB ions in the solution and the decreased interaction with the adsorption sites. Thermodynamical coefficients of the reaction were also calculated and are presented in Table 3. It was observed that the ΔG^0^ was negative at all temperatures, indicating a spontaneous reaction. Furthermore, the change in enthalpy was negative, confirming it as an exothermic reaction. The entropy value was also negative, signifying a decrease in molecular disorder at the interface of the adsorbent surface and the MB dye [60].

#### 3.2.5. Effect of the solid-to-liquid ratio on the adsorption reaction

To investigate the impact of the solid-to-liquid ratio (g mL^−1^) on the adsorption reaction, three experiments were conducted using the C5AgCAlg gel, 20 mL of 100 ppm MB solution at 20 °C and pH 7. The solid-to-liquid ratios of 0.5, 1.5, and 2 were investigated. Adsorption efficiencies were calculated as 95.68%, 96.72%, and 98.52%, respectively. As the quantity of adsorbent increased, the efficiency increased due to the increased adsorption surface area and sites [12].

#### 3.2.6. Effect of the initial MB concentration on the adsorption reaction

**Figure 8:**
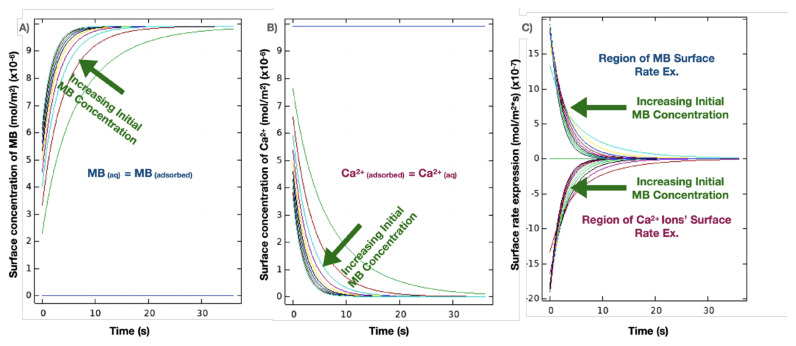
Calculated by changing the MB initial concentration, Ca^2+^ concentration on the surface (A), MB concentration (B), and surface rate expression (C).

To understand the effect of the initial MB concentration on the adsorption reaction, 0.5 g of the C5AgCAlg gels were stirred with 20 mL of 3 different solutions with different MB concentrations (5, 10, and 100 ppm) at 20 °C, pH 7, for 1 h. The calculated adsorption efficiency and q_e_ values are presented in Table 4. Increased contact between the MB ions and active adsorbent surfaces in the solution increased the adsorbent capacity in proportion to the initial concentration [12,61]. Moreover, in adsorption studies, the maximum adsorbent capacity can be reached based on the initial ion concentration, and this value increases with the increase in initial ion concentration. While the q_e_ value was expected to increase with an increasing ion concentration, the adsorption efficiency was expected to decrease. However, the study showed that increasing the MB ion concentration did not reduce the efficiency; on the contrary, it increased it. Computer-aided calculations were made to explain the increasing adsorption efficiency with increasing MB concentration. It was shown that the adsorption kinetics were compatible with pseudo-second-order, and a chemisorption-controlled reaction occurred. From this perspective, Eq. (17) was proposed for the adsorption reaction. It was assumed that the Ca^2+^ ionically bonded to the carboxyl groups of alginates were replaced by the MB^1+^ dissolved in the water. The concentration of MB^1+^ and Ca^2+^ on the surface and the reaction rate expression were calculated based on the constants and variables of the proposed reaction. These calculations are provided in Figure 8 and were done while considering the changes in the MB^1+^ concentrations. It was demonstrated that increasing the MB concentration did not affect the surface concentration when the reaction reached equilibrium. However, upon examination of the reaction kinetics, it became apparent that decreasing the concentration of MB resulted in a longer time required for the reaction to reach equilibrium. When operating with low concentrations of MB at 20 °C, pH 7, 0.5 g of gel, and 20 mL of solution, it was found that the time for the reaction to reach equilibrium was limited, and more time was required.


(17) $$Ca_{surface}^{2+}+2\;MB\;{ions}_{aq}^{+1}\to {Ca}_{aq}^{2+}+2MB\;{ions}_{surface}^{+1}$$

#### 3.2.7. Isotherm of the adsorption reaction

**Figure 9:**
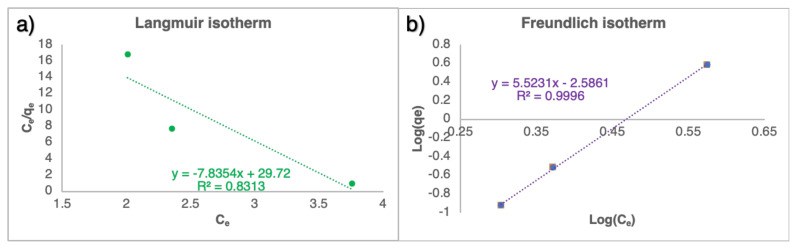
Compatibility of the adsorption reactions performed with C5AgCaAlg with the Langmuir (a) and Freundlich (b) isotherm models.

Experimental data were fitted with Langmuir and Freundlich Isotherm models to understand the relationship between adsorbent and adsorbate better. The C_e_/q_e_ was plotted against C_e_ in the Langmuir isotherm model, while log (q_e_) was plotted against log (C_e_) in the Freundlich model. The results are presented in Figure 9. The calculated model variables are also given in Table 5. It was understood that the adsorption reaction conformed the Freundlich model with an R^2^ value of 0.9996. Thus, adsorption occurred heterogeneously on multiple planes and surfaces [12,62]. However, the 1/n_f_ value in the Freundlich model was calculated to be greater than 1, which indicated that cooperative adsorption occurred [63], and the adsorbed adsorbate affected the adsorption of new adsorbate molecules [64].

### 3.3. Photocatalytic activity of the gels

A variety of techniques have been investigated for the removal of this substance, including sorption, photolysis (UV treatment), and photocatalysis. Sorption is a widely utilized method for the removal of MB that involves the adsorption of dye molecules onto solid adsorbents [65]. Photolysis involves the direct exposure of MB to UV light without the use of a catalyst [66]. Photocatalysis employs light energy to activate a photocatalyst, which then facilitates the degradation of MB [67]. The application of photocatalytic processes can result in the attainment of high removal rates. A comparative summary of the removal of MB by adsorption, photocatalysis, and photolysis (UV) is presented in Table S4. The process is based on the production of reactive species under UV irradiation with the assistance of a catalyst, which then initiates the degradation of the dye through an advanced oxidation processes (AOPs). AOPs have emerged as effective methods for degrading dyes such as MB. AOPs use highly reactive species such as hydroxyl radicals to oxidize organic contaminants [68]. Various AOPs, including ozone-based methods [68], Fenton processes [69], and UV-assisted techniques [70], have been investigated for the degradation of MB. The incorporation of materials with photocatalytic properties into gels is an effective method to remove contaminants from the waste solution under solar and UV irradiation. The current study aimed to purify the gels after their UV light-dark adsorption. Photocatalytic materials scatter rays of specific wavelengths due to their band gaps. Photocatalytic degradation is succinctly expressed in Eq. (18)–Eq. (28) [67]:


(18) $$Photocatalytic\;gel+irradiation\to Photocatalytic\;gel\;(h_{VB}^{+}+e_{CB}^{-})$$

(19) $$Pollutant\;material+irradiation\to Pollutant\;{material}^{*}$$

(20) $$Pollutant\;{material}^{*+}+Photocatalytic\;gel\to Pollutant\;material^{{}^{\circ}}+Photocatalytic\;gel\;(e_{CB}^{-})$$

(21) $$O_{2}+Photocatalytic\;gel\;(e_{CB}^{-})\to Photocatalytic\;gel+O_{2}^{{}^{\circ}-}$$

(22) $$O_{2}^{{}^{\circ}-}+H^{+}\to HO_{2}^{{}^{\circ}}$$

(23) $$2HO_{2}^{{}^{\circ}}\to O_{2}+H_{2}O_{2}$$

(24) $$H_{2}O_{2}+O_{2}^{{}^{\circ}-}+HO^{-}+HO^{{}^{\circ}}+O_{2}$$

(25) $$H_{2}O_{2}+e^{-}\to HO^{-}+HO^{{}^{\circ}}$$

(26) $$H_{2}O+Photocatalytic\;gel\;(h_{VB}^{+})\to Photocatalytic\;gel+HO^{{}^{\circ}}+H^{+}$$

(27) $$\begin{array}{l} HO^{-}+Photocatalytic\;gel\;(h_{VB}^{+})\to Photocatalytic\;gel+HO^{{}^{\circ}}\\ Pollutant\;material+Photocatalytic\;gel\;(h_{VB}^{+},O_{2}^{{}^{\circ}-},HO^{{}^{\circ}})\to \to \to \end{array}$$

(28) $$CO_{2}+NO_{2}+H_{2}O+other\;products$$

Ag NPs and other materials with overlapping conduction and valence bands (without a band gap) can emit radiation at specific wavelengths. This phenomenon is commonly referred to as SPR. The number of electrons present on the surface of these materials is responsible for regulating this radiation, which is dependent on their particle size and surface area due to the electron configuration of noble metals. The Ag NPs exhibited SPR, facilitating a photocatalytic process. Adsorption studies were carried out with 1 g of C5AgCAlg gel and 20-mL solutions containing 5, 10, and 100 ppm of MB at 20 °C, pH 7, 1 h in the dark, and 1 h under UV light. The adsorption efficiency calculated for solutions with three different concentrations is presented in Figure S6, and the UV diagrams of the solutions before and after adsorption are given in Figure S7. While the adsorption study performed with a solution containing 100 ppm reached 99.49% efficiency, the efficiency in studies using a solution containing 5 and 10 ppm was calculated as 78.24% and 76.44%, respectively. Even though 99.49% cleaning efficiency was achieved, the gels could not be cleaned entirely under UV light and were unsuitable for reuse, as explained in the next section. Even though the gels were suitable for reuse after adsorption with solutions with 5 and 10 ppm, the adsorption efficiencies were relatively low. Therefore, the study continued at these concentrations, increasing the solid from 1 to 4 g.

**Figure 10:**
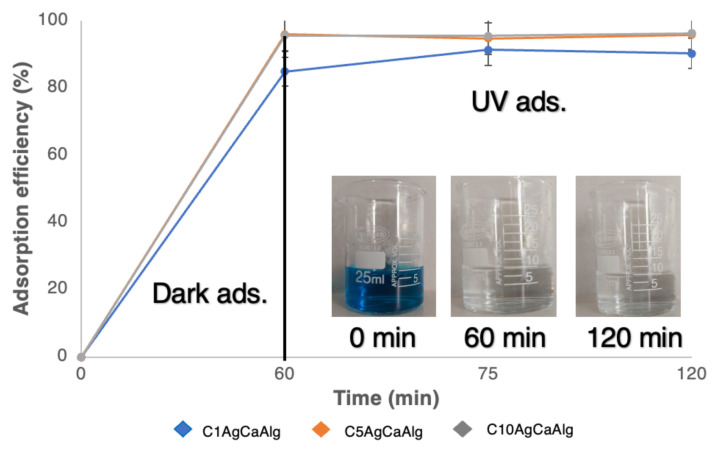
Adsorption efficiencies of the C1AgCaAlg, C5AgCaAlg, and C10AgCaAlg gels with 10 MB solutions in the 1 h dark and 1 h UV light environments (4 g of gel, 20 mL of 10 ppm solution at 20 °C, pH 7).

**Figure 11:**
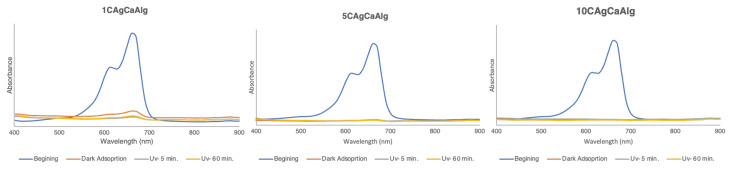
UV pattern of solutions before and after adsorption: C1AgCaAlg (a), C5AgCaAlg (b), and C10AgCaAlg (c).

An adsorption study was carried out with 4 g of the C1AgCaAlg, C5AgCaAlg, and C10AgCaAlg gels with 20 mL of 10 ppm solution at 20 °C, pH 7, 1 h in the dark and 1 h in UV environment. The adsorption efficiency calculated for solutions with the three gels is presented in Figure 10, and the UV diagrams of the solutions before and after adsorption are given in Figure 11. Efficiencies of 90.3%, 95.9%, and 96.32% were obtained for the C1AgCaAlg, C5AgCaAlg, and C10AgCaAlg gels, respectively, after a total of 2 h. As the Ag concentration in the gels increased, the UV adsorption efficiency also increased. In addition, the gels had been cleaned under these conditions, and it was possible to reuse them. Table 6 presents a summary of some photocatalytic degradation studies in the literature and the current study.

### 3.4. Characterization of the gels after adsorption

**Figure 12:**
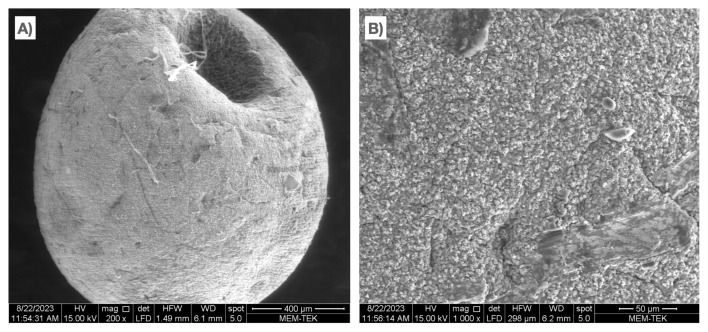
SEM images of the C5AgCaAlg gel after dark adsorption.

To understand the behavior of the gels under UV light, some physical characterization techniques were performed on the gels before and after stirring under UV light. Figure S8A shows the optical image of the cross-section of the C5AgCaAlg gel before adsorption in the dark environment. The color difference easily identifies the chitosan shell and calcium alginate core. Figure S8B illustrates the cross-section of the C5AgCaAlg gel obtained after adsorption with 10 ppm of MB (1 g of gel, 20 °C, pH 7, 1 h in the dark environment). Since the MB structure was positively charged, it was not found with chitosan due to its positively charged structure in the shell part, but it was found in the core part due to the negatively charged alginate structure. The cross-sectional image of the gel, whose adsorption was carried out under the same conditions and was completed under UV light for 1 h, is given in Figure S8C. Under UV light, the alginate core structure changed from blue to closer to its original color. It could be stated that MB dissociated from the gel’s structure and underwent disintegration under UV light. DSC analysis was conducted following dark adsorption and UV light adsorption of the 5CAgCaAlg gel. The peaks obtained are presented in Table 7. The sharp peak detected at approximately 190 °C was caused by the interference of the interaction between the Ca^2+^ and (CO_2_)^−^ ion and pertains to the disruption of the Egg-Box arrangement amidst the Ca^2+^ and alginate [12,78]. The sharp peak at 161.3 °C observed before UV adsorption was thought to indicate the interaction between the MB ions and carboxylate group ions. It can be thought that this peak arose from the disruption of the interaction between the positively charged MB ions and negatively charged alginate molecules. This peak at approximately 160 °C was not detected after UV adsorption, indicating that the MB within the structure was degraded under UV light. Figure S8D displays the gel image formed by the adsorption reaction when the solution contained 100 ppm of MB, and Figure S8E shows the adsorption result of the same gel under UV light. Although the adsorption efficiency was around 99%, the MB accumulated even in the chitosan layer and could not be removed from the structure with UV light. The excess amount of MB in the structure prevented the interaction between the Ag NPs and the UV light, and even if this were not the case, it was understood that the Ag concentration was insufficient to degrade the adsorbed MB. Moreover, the SEM images of the structure obtained after the dark adsorption of the C5AgCaAlg gel, using a 20-mL solution containing 100 ppm of MB at a temperature of 20 °C and pH 7, are presented in Figure 12. It can be seen that adsorption occurred on the entire surface, and the adsorption reaction did not cause a physical change on the surface of the material but preserved the structural integrity of the surface.

### 3.5. Cycle adsorption analysis of the gels

**Figure 13:**
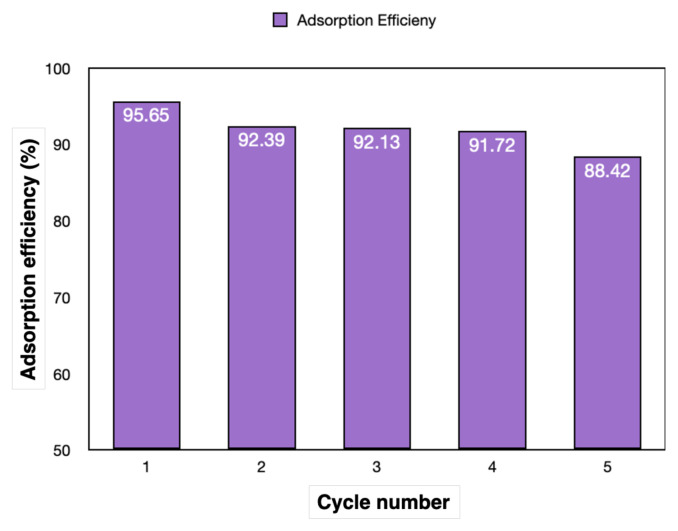
Cycle analysis of the C10AgCaAlg gel.

Cycle analysis was carried out to examine the reusability of the gels, and the adsorption efficiencies obtained as a result of the five cycles are shown in Figure 13. Cycle analysis was conducted by stirring 10 mL of a solution containing 10 ppm of MB with 2 g of the C10AgCAlg gel at pH 7, 20 °C, for 1 h in the dark and 1 h under UV light. The gels exhibited efficiency exceeding 90% in the initial four cycles and 88% in the cycle 5. Through cycle analysis, it was determined that the gels could be purified under UV light without any additional deadsorption process and that the structure retained its stability.

### 3.6. Energy consumption

One of the primary factors used to evaluate the efficacy of wastewater treatment systems is the system’s energy consumption (EC): and the EC rate of the system was determined using Eq. (29) [79]:


(29) $$\text{EC}=\frac{P\times t}{V\times \text{log}(\frac{C_{0}}{C_{e}})}\times \frac{1000}{60}$$

In this context, the variables V, t, and P represent the volume of the MB solution, the treatment time, and the input power (kW), respectively. The obtained results show that 6.972 kWh L^−1^ energy was required for photocatalytic MB degradation.

### 3.7. Long-term stability of the gels

**Figure 14:**
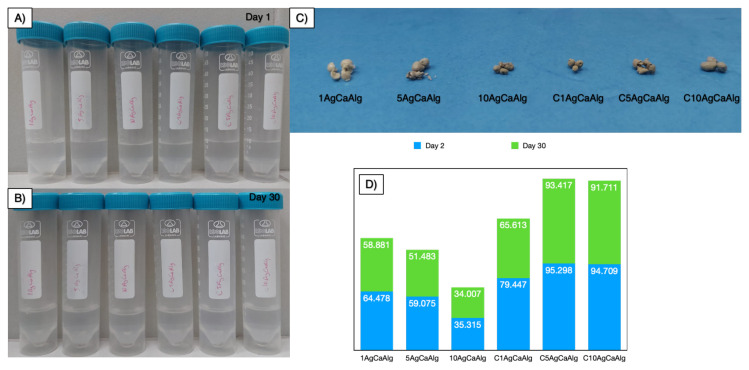
Storage of the gels on days 1 (A) and 30 (B), optical images of the gels on day 30 (C), and weight stability of the gels on day 30 (D).

Carboxyl groups in the structure of alginate can be protonated in low pH systems and lose their stable structure, and coating with chitosan, which has high low pH stability, increased the low pH stability of the polyelectrolyte gels formed [12]. All the gels, uncoated and coated with chitosan, were kept in 0.1 M HNO_3_ for 30 days, and the weight loss in their structures was observed. Storage images of the gels at the end of days 1 and 30 are given in Figures 14A and 14B, respectively. The gels were removed from the solution at the end of day 30, and their images were taken and are shown in Figure 14C. While the gels coated with chitosan retained their structural integrity, disintegration was observed in those not coated with chitosan. Additionally, the weight conservation in the structure of the gels is given in Figure 14D. At the end of day 1, the alginate gels released water in the low pH environment, and some weight loss due to shrinkage was observed in the six different gels. The gels began to disintegrate at the end of day 2, and how much of their weight could be maintained at the end of day 2 was examined. The chitosan-coated gels were able to maintain between 80% and 95% stability, while those without chitosan coating were able to maintain between 35% and 65% stability. At the end of day 30, there was a 1% to 6% change in the gels compared to day 2. Considering the change in all the gels, it was found that coating with chitosan increased the pH stability of the calcium alginate gels and allowed them to maintain their stable structure.

### 3.8. Cytotoxicity of the gels

**Figure 15:**
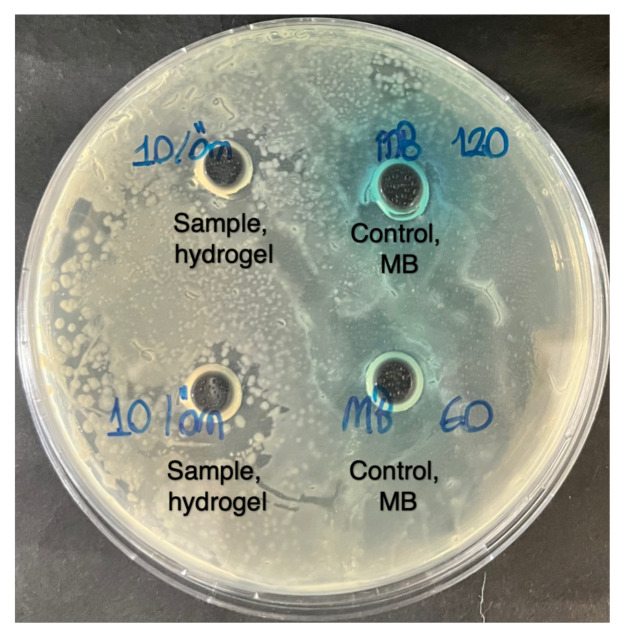
Toxicity analysis of the treated MB solution (120 and 60 ppm) and hydrogel samples.

The water samples analyzed showed no evidence of bacterial inhibition during the toxicity test (see Figure 15). However, the water samples treated with MB, used as the control group, exhibited a noteworthy bacterial inhibitory effect with both 120 ppm (with an average inhibition of 20.00 ± 1.63%) and 60 ppm (with an average inhibition of 16.67 ± 2.36%).

## Conclusion

The objective of this study was to investigate the effects of chitosan-coated calcium alginate gel containing Ag NPs on MB adsorption and photocatalytic degradation. It was demonstrated that varying concentrations of Ag NPs enhanced the adsorption efficiency by increasing the surface roughness of the gel. While the C1AgCaAlg gel reached equilibrium with an efficiency of 90.59% within 60 min, the C5AgCaAlg and C10AgCaAlg gels reached equilibrium with efficiencies of 95.67% and 96.17%, respectively, within the same time frame. An increase in the concentration of Ag NPs resulted in a reduction in the equilibrium time and an enhancement in efficiency. The q_e_ value was calculated as 3.62 mg g^−1^ for the C1AgCaAlg gel, 3.82 mg g^−1^ for the C5AgCaAlg gel, and 3.84 mg g^−1^ for the C10AgCaAlg gel. The adsorption kinetics were consistent with the pseudo-second-order model, indicating that chemical adsorption (chemisorption) was the dominant process. Moreover, the application of a chitosan coating did not result in a notable impact on the adsorption efficiency and kinetics. The degradation capacity of MB by the Ag NPs-containing gels was significantly enhanced under UV light with an increasing Ag NPs concentration. The C1AgCaAlg gel demonstrated a notable efficacy in degrading MB under UV light, with a degradation efficiency of 92.5%. The C5AgCaAlg and C10AgCaAlg gels exhibited enhanced degradation efficiency, reaching 95.2% and 97.8%, respectively. The photocatalytic degradation mechanism indicated that the MB was separated from the gel under UV light and entered the degradation process. Cyclic analyses demonstrated that the gels could be reused with high efficiency for up to five cycles. The structural integrity of the chitosan-coated gels was maintained at a rate of between 80% and 95% in acidic media (pH 2), whereas this rate decreased below 60% for the uncoated gels. In conclusion, the chitosan-coated calcium alginate gels containing Ag NPs were demonstrated to exhibit high efficiency, reusability, and long-term stability in the adsorption and photocatalytic degradation of MB.

## Supplemantary information

Figure S1 Experimental scheme.

**Table S1:** Parameters and levels of experiments.

Parameter	Levels			
Time (min)	0–180			
Chitosan coating	−		+	
Temperature (°C)	5	20	50	80
Initial MB concentration (ppm)	5	10	100	
Amount of adsorbent (g)	1	2	4	

Figure S2 Optical images of the 1AgCaAlg (a), 5AgCaAlg (d), 10AgCaAlg (g), C1AgCaAlg (b, c), C5AgCaAlg (e, f), and C10AgCaAlg (h, i) gels.

**Table S2:** Model constants for simulation.

Constants	Definition	Value
K _eq0_	Equilibrium constant	0.25
C _H2O_	Water concentration (mol m ^−3^)	55,600
V _f_	Volumetric feed inlet (m ^3^ s ^−1^)	1
V _r_	Reactor volume (m ^3^)	1
A _r_	Surface area (m ^2^)	20,000

Figure S3 UV spectra (a), and optical absorption coefficient vs. the photon energy (b) of the chitosan-coated gels.

**Table S3:** Parameters and levels analyzed for their impact on the adsorption reaction.

Parameter	Levels			
Gel	C1AgCAlg	C5AgCAlg		C10AgCAlg
Initial MB concentration (ppm)	5	10		100
Amount of adsorbent (g)	1		4	

Figure S4 Surface roughness of the gels: a) C1AgCaAlg, b) C5AgCaAlg, and c) C10AgCaAlg.

**Table S4:** Comparison of MB removal by adsorption, photocatalysis, and photolysis (UV) [1–4].

	Sorption	Photocatalysis	Photolysis
Mechanism	Involves physical or chemical interactions between the dye and adsorbent surfaces, leading to dye retention.	Utilizes light energy to generate reactive species (e.g., hydroxyl radicals) that decompose the dye.	Relies on the energy from light to break chemical bonds in the dye molecules directly.
Efficiency factors	Influenced by adsorbent type, surface area, contact time, and initial dye concentration.	Affected by catalyst type, light intensity, wavelength, and reaction time.	Dependent on light intensity, wavelength, and exposure duration; often less efficient than other methods.
Advantages	Simple operation; can be effective with low-cost natural materials; can be used in batch processes.	High efficiency for dye degradation; can mineralize organic pollutants completely; reusable catalysts possible.	Straightforward process that requires minimal setup; no need for additional chemicals or catalysts.
Limitations	May lead to saturation of adsorbent; requires regeneration or disposal of spent adsorbents; potential for secondary pollution if byproducts are formed.	Requires specific conditions (e.g., UV light); catalyst deactivation can occur over time; may produce intermediate byproducts.	Generally, less efficient than other methods; may require longer treatment times and can lead to incomplete degradation.

Figure S5 Adsorption efficiency (a), adsorbent capacity (b), adsorption reaction compatibility with the pseudo-first-order (c), and second-order (d) of 10AgCaAlg (1 g of gel and 20 mL of 100 ppm MB solution at 20 °C, pH 7, 2 h).

Figure S6 Adsorption efficiencies of the C5AgCaAlg gel with 5, 10, and 100 ppm MB solutions at 1 h dark and 1 h UV light environment (1g of C5AgCAlg gel, 20 mL of solution, 20 °C, pH 7).

Figure S7 UV patterns of the solutions before and after adsorption, with 5 ppm (a), 10 ppm (b), and 100 ppm (c).

Figure S8 Cross-sectional image of the C5AgCaAlg gel (a), C5AgCaAlg gel after dark adsorption (with 10 ppm) (c), C5AgCaAlg gel after UV adsorption (with 10 ppm) (d), C5AgCaAlg gel after dark adsorption (with 100 ppm), and C5AgCaAlg gel after UV light adsorption (with 100 ppm).

Supplemental information references1

Jaramillo-FierroX
CuencaG

Enhancing Methylene Blue Removal through Adsorption and Photocatalysis—A Study on the GO/ZnTiO3/TiO2 Composite
International Journal of Molecular Sciences
2024
25
8
4367
10.3390/ijms25084367
38673952
PMC110498372

JuzsakovaT
SalmanAD
AbdullahTA
RasheedRT
ZsirkaB


Removal of Methylene Blue from Aqueous Solution by Mixture of Reused Silica Gel Desiccant and Natural Sand or Eggshell Waste
Materials
2023
16
4
1618
10.3390/ma16041618
36837246
PMC99651023

FarouqR

Coupling Adsorption-Photocatalytic Degradation of Methylene Blue and Maxilon Red
Journal of Fluorescence
2022
32
4
1381
1388
10.1007/s10895-022-02934-1
35384544
PMC92703134

UlfaM
Al AfifH
SaraswatiTE
BahrujiH

Fast Removal of Methylene Blue via Adsorption-Photodegradation on TiO2/SBA-15 Synthesized by Slow Calcination
Materials
2022
15
16
5471
10.3390/ma15165471
36013608
PMC9409962

## Figures and Tables

**Table 1:** Descriptions of the hydrogels, chitosan coating, and the amount of Ag NPs in the gels.

Material	Amount of sodium alginate solution (mL)	Chitosan coating	Amount of Ag NPs (g)
1AgCaAlg	20	−	0.2
5AgCaAlg	20	−	1
10AgCaAlg	20	−	2
C1AgCAlg	20	+	0.2
C5AgCAlg	20	+	1
C10AgCAlg	20	+	2

**Table 2:** The constants of pseudo-first and second-order adsorption reaction.

Gels	Pseudo-first-order			Pseudo-second-order		
	k_1_	q_e calculated_ (mg g^−1^)	R^2^	k_2_	q_e calculated_ (mg g^−1^)	R^2^
C1AgCaAlg	0.0239	0.511	0.4364	0.076	3.699	0.9995
C5AgCaAlg	0.0197	0.317	0.4891	0.096	3.912	0.9991
C10AgCaAlg	0.0245	0.263	0.629	0.065	3.910	0.9997

**Table 3:** Adsorption efficiency for a variety of temperatures and the thermodynamic constants of the adsorption reaction.

Temperature (°C)	Adsorption efficiency (%)	ΔG° (kJ mol^−1^)	ΔH° (kJ mol^−1^)	ΔS^o^ (j mol^−1^ K^−1^)
20	96.36	−9.67	−24.77	−63.92
50	86.12	−6.76	−24.77	−63.92
80	76.17	−5.45	−24.77	−63.92

**Table 4:** Adsorption efficiency and q_e_ values calculated with varying initial MB concentrations.

Initial MB concentration (ppm)	Adsorption efficiency (%)	q_e_ (mg g^−1^)
5	59.77	0.12
10	76.44	0.30
100	96.24	3.85

**Table 5:** The constants of Langmuir and Freundlich isotherm models.

Langmuir model			Freundlich model		
q_m_	k_L_	R^2^	n_f_	k_F_	R^2^
0.0033	−38.25	0.8313	0.83	0.0025	0.9996

**Table 6:** Some studies investigating the use of photocatalytic degradation for the removal of dyes from wastewater.

Material	Pollutant	Time (min)	Degradation efficiency (%)	Source
Sodium alginate/hydroxyethyl methacrylate-graphene oxide decorated ZnO	MB	150	94.5	[71]
Alginate beads-kaolinite/graphitic carbon nitride	Brilliant green	90	97	[72]
Iron (III)-alginate	Tetracycline	120	97.3	[73]
Calcium alginate-CuO/graphitic carbon nitride	MB	60	86.26	[74]
Ag_3_PO_4_/Co_3_(PO_4_)_2_/graphitic carbon nitride	Tetracycline	120	88	[75]
Chitosan- N, S-doped TiO_2_/CS	Tetracycline	20	91	[76]
TiO_2_–ZnO/CS–Gr	Tetracycline	180	97.2	[77]
Calcium alginate/chitosan-Ag NPs	MB	120	96.32	This study

**Table 7:** Peaks obtained from the DSC analysis of the C5AgCaAlg gel before and after UV adsorption.

	Temperature (°C)	Peak type	Thermodynamic of the peak
Before UV ads.	161.3	Sharp	Exo.
	190	Sharp	Exo.
After UV ads	123.9	Board	Exo.
	198.6	Sharp	Exo.
