# Supplementary figures and images for: Synthesis of low pH stable, highly photocatalytic alginate hydrogels for methylene blue removal from wastewater
Source: Turk J Chem. 2024 Dec 8;49(2):154–75. doi: 10.55730/1300-0527.3719 (PMC12068674; doi:10.55730/1300-0527.3719)

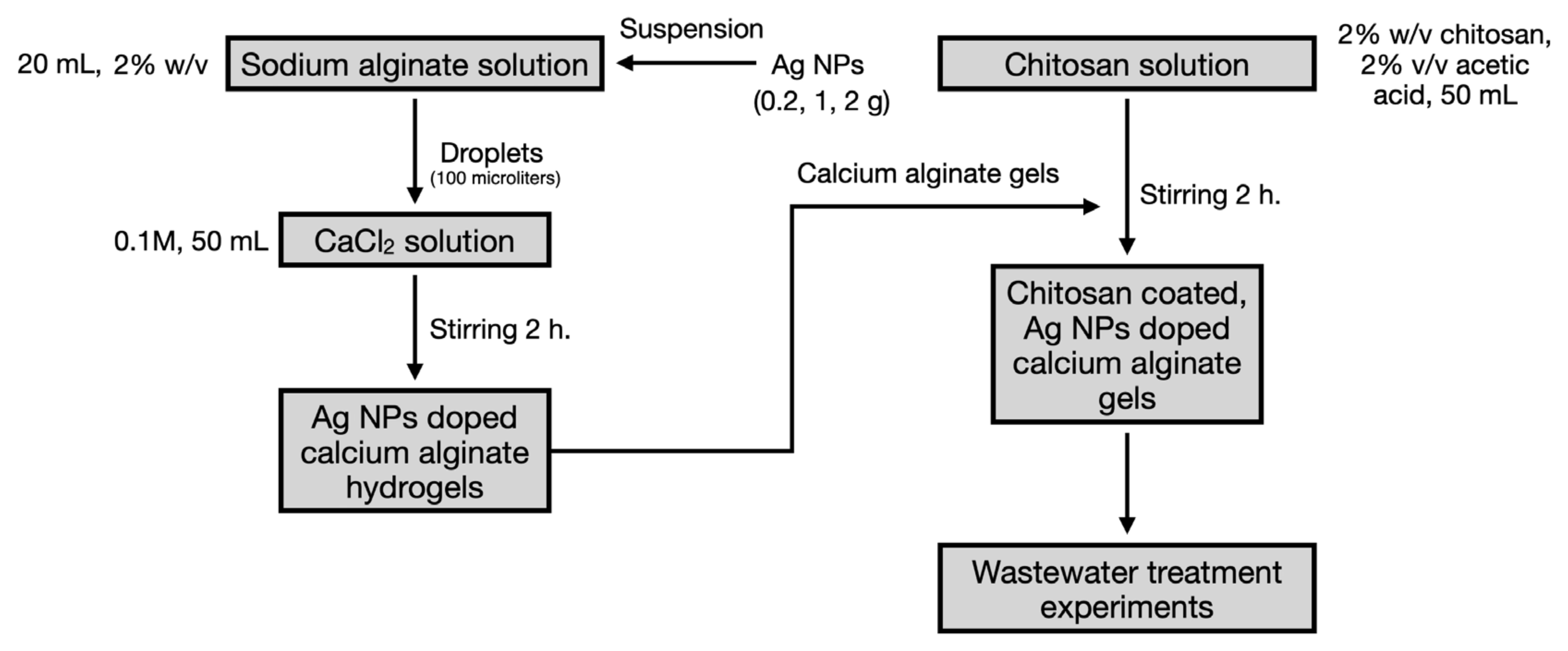

Supplement: Figure S1 — Experimental scheme. [file tjc-49-02-154s1.tif]

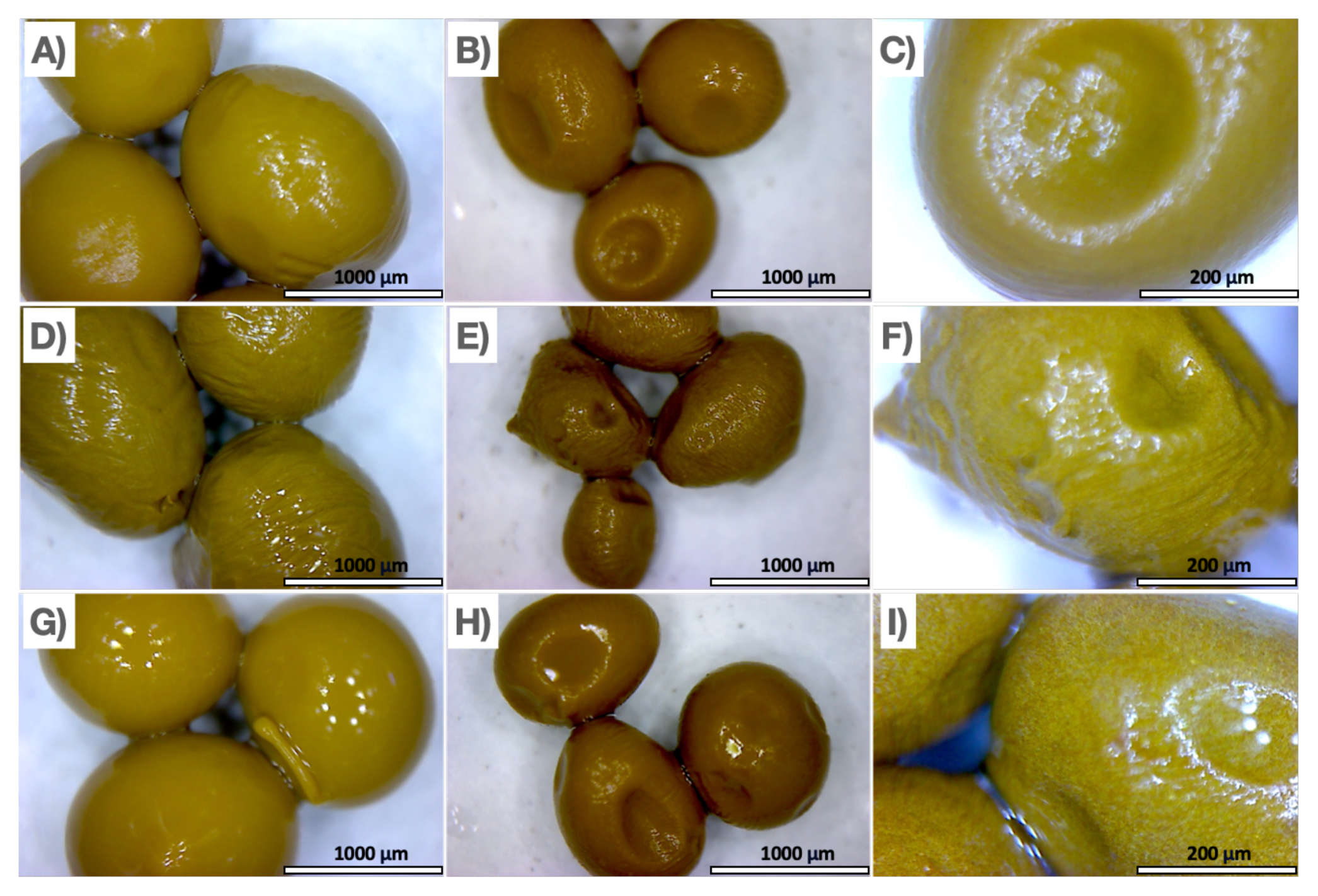

Supplement: Figure S2 — Optical images of the 1AgCaAlg (a), 5AgCaAlg (d), 10AgCaAlg (g), C1AgCaAlg (b, c), C5AgCaAlg (e, f), and C10AgCaAlg (h, i) gels. [file tjc-49-02-154s2.tif]

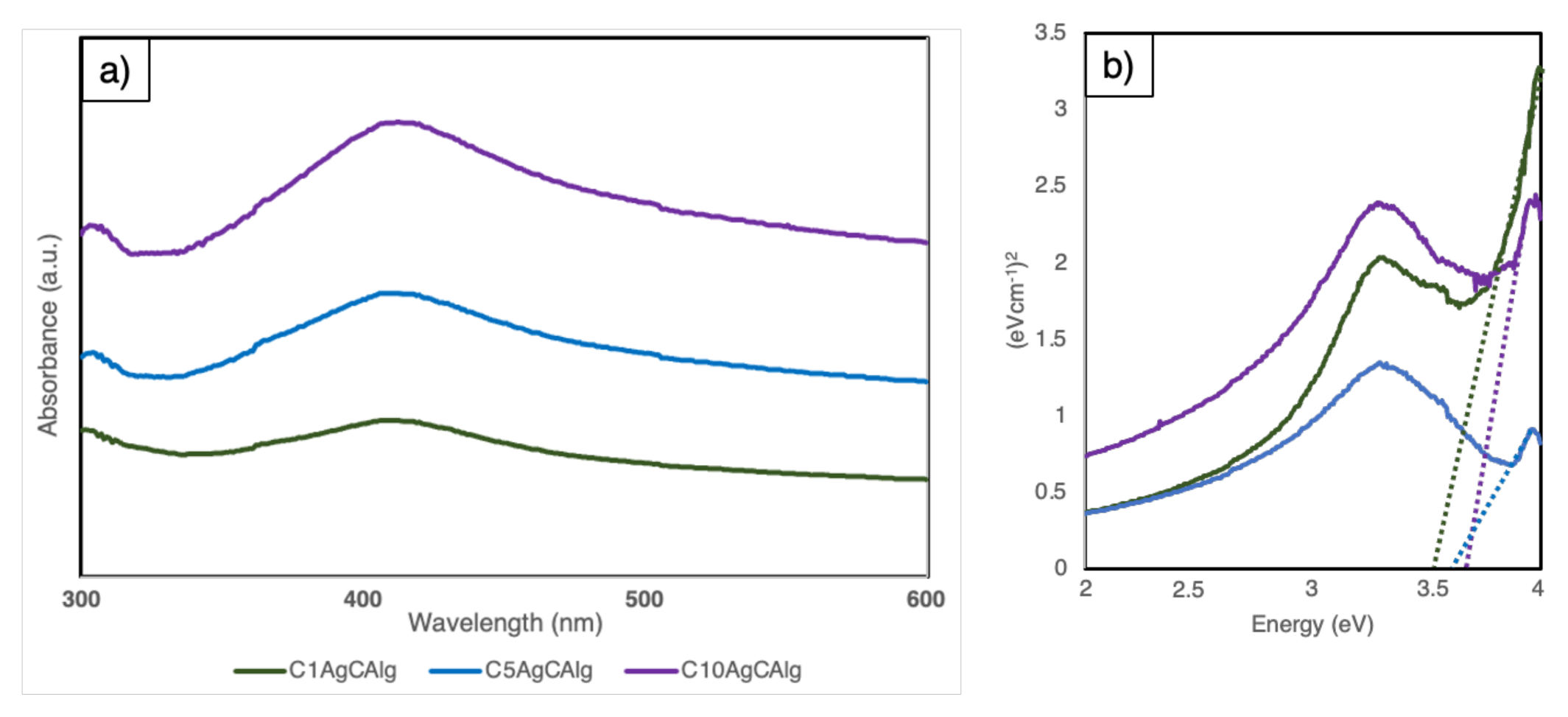

Supplement: Figure S3 — UV spectra (a), and optical absorption coefficient vs. the photon energy (b) of the chitosan-coated gels. [file tjc-49-02-154s3.tif]

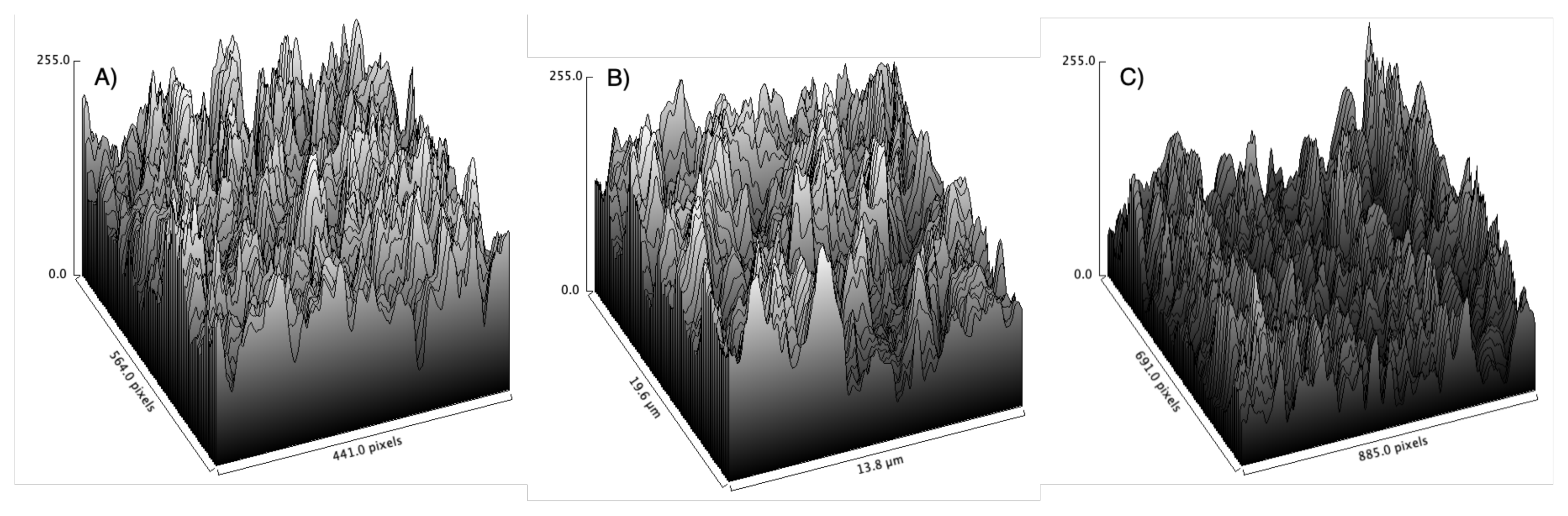

Supplement: Figure S4 — Surface roughness of the gels: a) C1AgCaAlg, b) C5AgCaAlg, and c) C10AgCaAlg. [file tjc-49-02-154s4.tif]

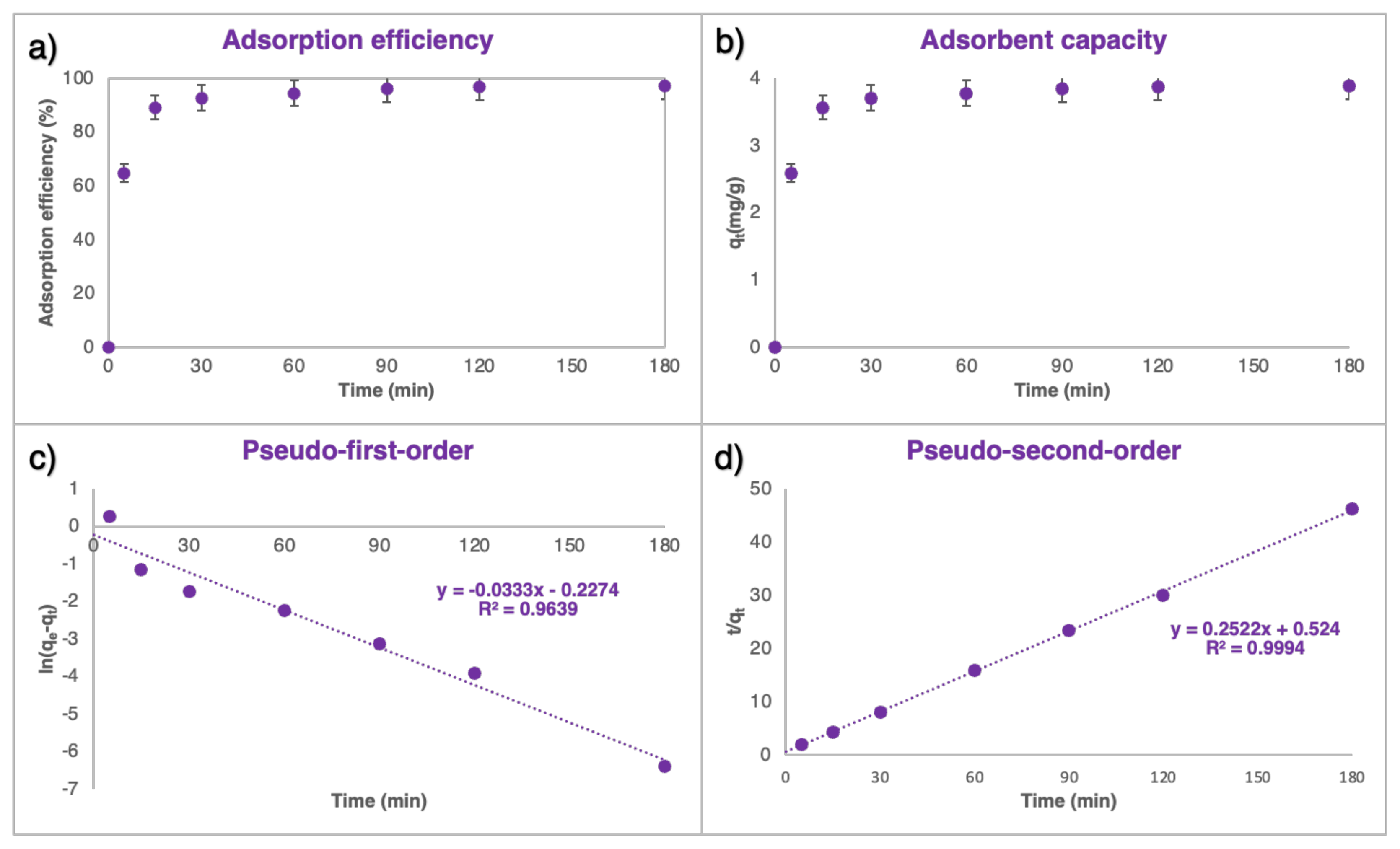

Supplement: Figure S5 — Adsorption efficiency (a), adsorbent capacity (b), adsorption reaction compatibility with the pseudo-first-order (c), and second-order (d) of 10AgCaAlg (1 g of gel and 20 mL of 100 ppm MB solution at 20 °C, pH 7, 2 h). [file tjc-49-02-154s5.tif]

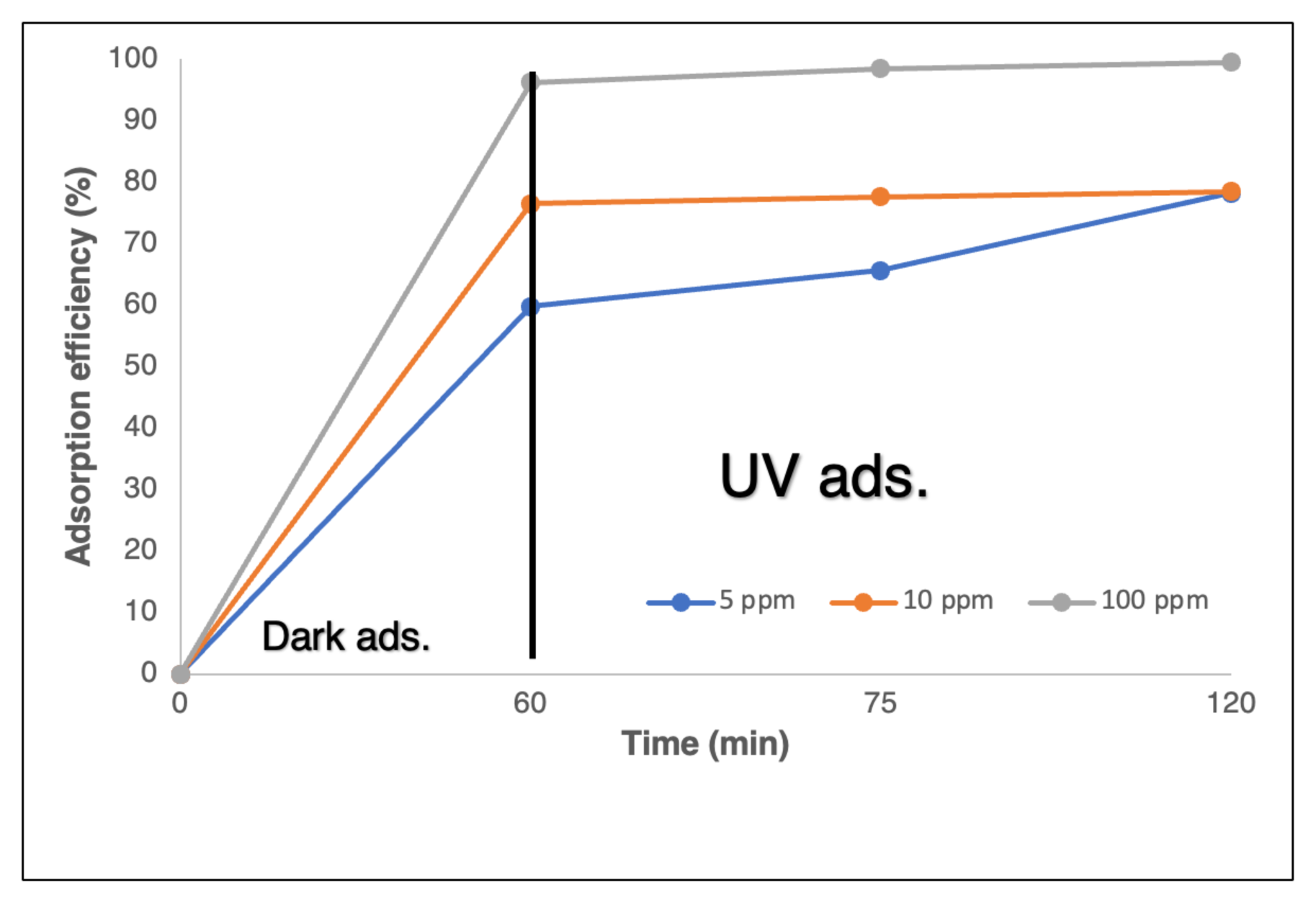

Supplement: Figure S6 — Adsorption efficiencies of the C5AgCaAlg gel with 5, 10, and 100 ppm MB solutions at 1 h dark and 1 h UV light environment (1g of C5AgCAlg gel, 20 mL of solution, 20 °C, pH 7). [file tjc-49-02-154s6.tif]

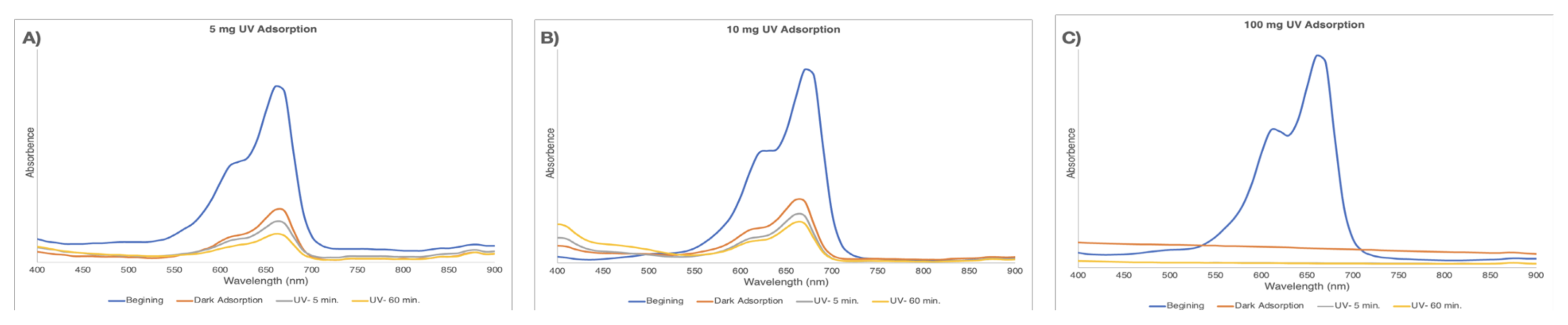

Supplement: Figure S7 — UV patterns of the solutions before and after adsorption, with 5 ppm (a), 10 ppm (b), and 100 ppm (c). [file tjc-49-02-154s7.tif]

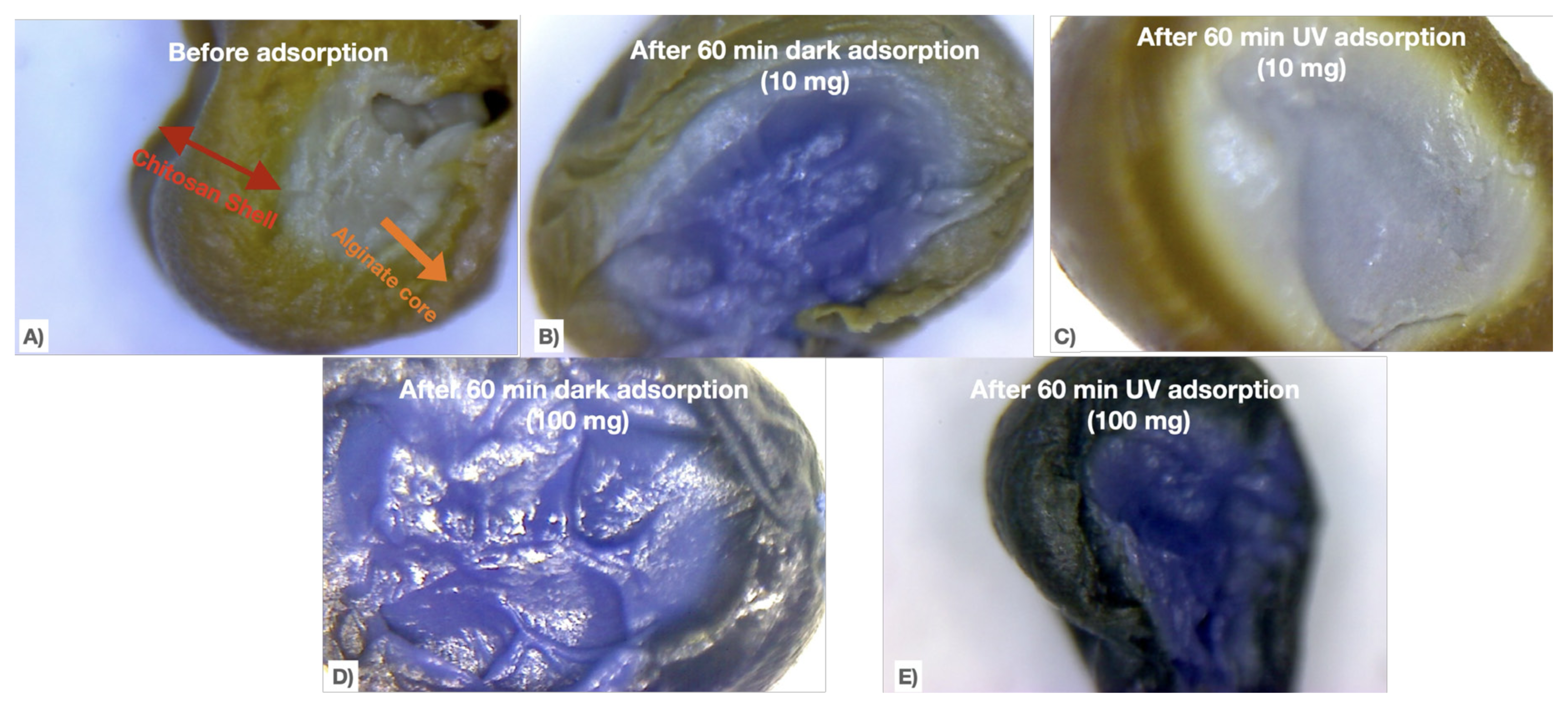

Supplement: Figure S8 — Cross-sectional image of the C5AgCaAlg gel (a), C5AgCaAlg gel after dark adsorption (with 10 ppm) (c), C5AgCaAlg gel after UV adsorption (with 10 ppm) (d), C5AgCaAlg gel after dark adsorption (with 100 ppm), and C5AgCaAlg gel after UV light adsorption (with 100 ppm). [file tjc-49-02-154s8.tif]
